# QuickStats

**Published:** 2013-08-02

**Authors:** Susan L. Lukacs, Cathy Duran

**Figure:**
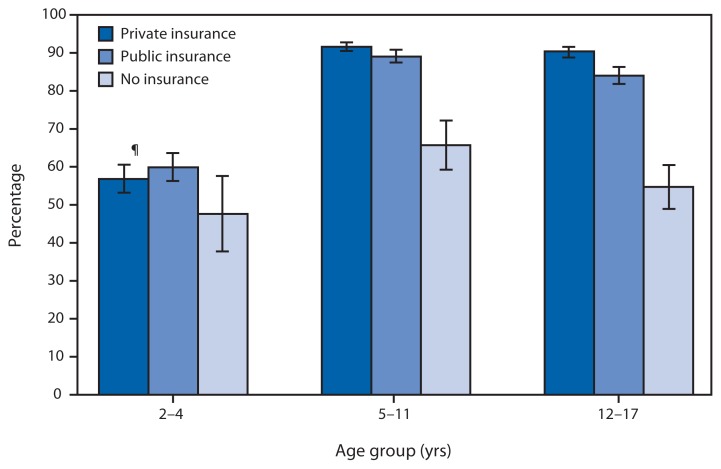
Percentage of Children Aged 2–17 Years With a Dental Visit in the Past Year,* by Age Group and Health Insurance Status^†^ — National Health Interview Survey,^§^ United States, 2011 * Children were identified as having a dental visit in the past year by asking parents, “About how long has it been since your child last saw a dentist?” Parents were directed to include all types of dentists, including orthodontists, oral surgeons, and all other dental specialists, as well as dental hygienists. ^†^ Children with health insurance might or might not have dental coverage. Children with both public and private insurance coverage are placed in the private insurance category. Public health insurance for children consists mostly of Medicaid, but also includes Medicare, the Children’s Health Insurance Programs, and Tricare. ^§^ Estimates were based on household interviews of a sample of the noninstitutionalized, civilian U.S. population and are derived from the National Health Interview Survey sample child component. ^¶^ 95% confidence interval.

In 2011, the percentage of children aged 5–11 years and aged 12–17 years who had a dental visit in the past year was higher for children from families with private or public health insurance compared with children from families with no health insurance. For children aged 5–11 years, 92% with private insurance, 89% with public insurance, and 66% with no health insurance had a dental visit in the past year. The percentages were similar for children aged 12–17 years: 90% for those with private insurance, 84% for those with public insurance, and 55% for those without insurance. Fewer than two thirds of children aged 2–4 years had a dental visit in the past year, regardless of insurance status.

**Source:** Federal Interagency Forum on Child and Family Statistics. America’s children: key national indicators of well-being, 2013. Washington, DC: US Government Printing Office; 2013. Available at http://childstats.gov.

